# Risk Factors for Addictive Behaviors: A General Overview

**DOI:** 10.3390/ijerph19116583

**Published:** 2022-05-28

**Authors:** Omar Enzo Santangelo, Sandro Provenzano, Alberto Firenze

**Affiliations:** 1 Regional Health Care and Social Agency of Lodi, ASST Lodi, 26900 Lodi, Italy; omarenzosantangelo@hotmail.it; 2 Local Health Unit of Trapani, ASP Trapani, 91100 Trapani, Italy; 3 Department of Health Promotion, Mother and Child Care, Internal Medicine and Medical Specialties (ProMISE) “G. D’Alessandro”, University of Palermo, Via del Vespro, 133, 90127 Palermo, Italy; alberto.firenze@unipa.it

Many people around the world have behaviors that are recognized as addictive behaviors, usually not causing significant health consequences except in a few cases, which consist typically of a low percentage of people who may develop addictive behavioral disorders that may be associated with functional impairment and distress [1]. The term “addiction” is common in today’s society, despite the lack of a single definition [2], although the Diagnostic and Statistical Manual of Mental Disorders, Fifth Edition (DSM-5) states that the term can be used to describe severe substance use disorders [3].

Very interesting and plausible is the definition provided by the American Society of Addiction Medicine which does not identify “addiction” with the sole use of substances/drugs but also as the assumption of compulsive behaviors that continue despite the harmful consequences for the individual, identifying the biological and genetic dimensions of addiction, as well as the environmental and lifestyle dimensions. Specifically, the definition of the American Society of Addiction Medicine is as follows: “Addiction is a treatable, chronic medical disease involving complex interactions among brain circuits, genetics, the environment, and an individual’s life experiences. People with addiction use substances or engage in behaviors that become compulsive and often continue despite harmful consequences. Prevention efforts and treatment approaches for addiction are generally as successful as those for other chronic diseases [4].”

An activity, substance, object, or behavior that becomes the main focus of a person’s life excluding other activities, or that has begun to harm the individual or others physically, mentally, or socially can be considered addictive behavior, a person can become addicted or compulsively obsessed with anything [5]. Some researchers suggest that there may be similarities between physical dependence on various chemicals, and psychological dependence on activities such as eating disorders. It is thought that these behavioral activities may produce betaendorphins in the brain causing the person to remain engaged in activity to achieve this feeling of well-being and euphoria, risking entering a cycle of addiction [6]. Thus, the person may risk becoming physically dependent on the chemicals in their brain, leading to the continuation of the behavior even though it can have negative health or social consequences [5,6,7].

New addictions are taking hold in recent years, together with the classic addictions to drugs or substances, for example, smoking, alcohol and drugs, also the so-called non-substance addiction (or behavioral addiction) that affect addictions such as gambling, food addiction, or the internet addiction [5].

Among young people, the use of videogames and the internet is widespread, generally it is a healthy hobby for most users. However, in recent years there has been a growing global recognition among public health professionals and academics that particular video game models can lead to marked impairment of personal, family, social, educational, occupational, or other important areas of functioning and psychological distress for a significant minority of players. In fact, often not enough attention is given to the time of play or internet use with the risk of exclusion of other daily activities, and therefore with the possibility of changes in their physical or psychological health and social functioning [1,8].

Gambling in many countries and jurisdictions is considered a form of entertainment, characterized by betting mechanisms and monetization features. Like gambling, repetitive gambling behavior can potentially lead to gambling disturbances associated with distress or impairment [9]. The increasing availability of the internet is associated not only with obvious benefits for users and societies, but also with documented cases of overuse that often have adverse health consequences [9]. The subsequent convergence in recent years between gaming and gambling aided by the availability of the internet has led to the frequent migration from games to gambling with related health problems. Health problems associated with gambling behavior are not limited to gambling disorder, but also include other health aspects, such as, insufficient physical activity, unhealthy diet, vision or hearing problems, musculoskeletal problems, sleep deprivation, and associated health conditions such as depression and venous thromboembolism disorders [10]. The prevalence of “problem gambling,” which is a proxy measure for the prevalence of gambling disorder among adults, ranges between 0.1% and 5.8% [10]. The harms caused by gambling are significant according to some researchers comparable to the harms due to depression and alcohol use disorders [9,10]. The damage negatively impacts the players/gamblers themselves, their families, and the community [8,9,10]. Gambling disorder was recently included as a new condition in the 11th revision of the International Classification of Diseases (ICD-11) approved by the 72nd World Health Assembly (WHA72) in 2019.

The term “food addiction” has been used in combination with specific eating behaviors to describe an abnormal pattern of excessive consumption [11]. According to Pursey et al., the average prevalence of food addiction is around 20% but, more importantly, the identification and possible treatment of food addiction symptoms at a young age could prevent the carryover of food addiction trends from infancy to childhood to adulthood, just like the increased risk of adult obesity is associated with childhood obesity [12].

The use of psychoactive drugs without medical supervision is associated with significant health risks and can lead to the development of drug use disorders. Drug use disorders, particularly when untreated, increase morbidity and mortality risks for individuals, and can trigger substantial suffering and lead to impairment in personal, family, social, educational, occupational, or other important areas of functioning. Drug use disorders are associated with significant costs to society due to loss of productivity, premature mortality, increased health care expenditure, and costs related to criminal justice, social welfare, and other social consequences [13]. About 270 million people (or about 5.5% of global population aged 15–64) had used psychoactive drugs in the previous year and about 35 million people are estimated to be affected by drug use disorders (harmful patterns of drug use or drug dependence). It is estimated that about 0.5 million deaths annually are attributable to drug use with about 350,000 male and 150,000 female deaths. Opioid-related deaths, largely due to synthetic opioids, have recently changed the mortality trends in some high-income countries. More than 42 million years of healthy life loss (DALY) were attributable to drug use in 2017; that is about 1.3% of the global burden of disease. It is estimated that worldwide there are almost 11 million people who inject drugs, of whom 1.4 million live with HIV and 5.6 million with hepatitis [13].

Over 7 million deaths per year are the result of direct tobacco use, while approximately 1.2 million are the result of exposure to secondhand smoke from non-smokers [14]. Tobacco smoking, including passive smoking, is a major risk factor attributable to 6% of global disability-adjusted life years [15], but in the absence of drastic control measures the numbers are set to grow further [14].

Another worldwide problem of great interest for public health is the harmful use of alcohol, about 3 million deaths per year worldwide are attributable to the harmful use of alcohol, 5.3% of total deaths. It is also a risk factor in over 200 disease and injury conditions [16]. Alcohol consumption causes death and disability relatively early in life. In people aged 20 to 39, about 13.5% of total deaths are attributable to alcohol, often alcoholism is also associated with smoking, anxiety, and depression, increasing the global burden of disease, particularly in the young population [16,17,18].

Certainly there are other addictive behaviors, in this editorial we have tried to give an overview of those who have a greater burden of disease and to highlight the new addictive behaviors that are taking place and which are definitely worth keeping an eye on.

Of great importance for the control and management of these situations could be the training and information campaigns for the target populations, educating the students from school age on the risks of these behaviors and on the socio-economic costs that derive from them, above all because these factors typically affect a young population. Even the socio-economic condition can be an important risk factor for the onset of these addictions, it would be advisable to implement prevention interventions especially in the most disadvantaged social classes.
